# Characterization and predictive risk scoring of long COVID in a south indian cohort after breakthrough COVID infection; a prospective single centre study

**DOI:** 10.1186/s12879-023-08600-6

**Published:** 2023-10-09

**Authors:** Pranav Nair, Chithira V Nair, Kiran G Kulirankal, Elizabeth M Corley, Fabia Edathadathil, Georg Gutjahr, Merlin Moni, Dipu T Sathyapalan

**Affiliations:** 1 Department of Radiation Oncology, Amrita Institute of Medical Science and Research Centre, Amrita Vishwa Vidyapeetham, Kochi, Kerala India; 2 Division of Infectious Diseases, Department of General Medicine, Amrita Institute of Medical Science and Research Centre, Amrita Vishwa Vidyapeetham, Kochi, Kerala India; 3 Department of Internal Medicine, Weill Cornell, New York, USA; 4 Department of Infection Control and Epidemiology, Amrita Institute of Medical Science and Research Centre, Amrita Vishwa Vidyapeetham, Kochi, Kerala India; 5 Center for Research in Analytics and Technologies for Education (CREATE), Amrita Vishwa Vidyapeetham, Amritapuri, Kollam, Kerala India; 6 Amrita Institute of Medical Science and Research Centre, Amrita Vishwa Vidhyapeetham, Kochi, 682041 Kerala India

**Keywords:** Long COVID, Post COVID syndrome, Breakthrough infections

## Abstract

**Background:**

With the World Health Organization (WHO) declaring an end to the COVID-19 pandemic, the focus has shifted to understanding and managing long-term post-infectious complications. “Long COVID,“ characterized by persistent or new onset symptoms extending beyond the initial phase of infection, is one such complication. This study aims to describe the incidence, clinical features and risk profile of long COVID among individuals in a South Indian cohort who experienced post-ChAdOx1 n-Cov-2 vaccine breakthrough infections.

**Methods:**

A single-centre hospital-based prospective observational study was conducted from October to December 2021. The study population comprised adult patients (> 18 years) with a confirmed COVID-19 diagnosis who had received at least a single dose of vaccination. Data was collected using a specially tailored questionnaire at week 2, week 6, and week 12 post-negative COVID-19 test. A propensity score based predictive scoring system was developed to assess the risk of long COVID.

**Results:**

Among the 414 patients followed up in the study, 164 (39.6%) reported long COVID symptoms persisting beyond 6 week’s post-infection. The presence of long COVID was significantly higher among patients above 65 years of age, and those with comorbidities such as Type II Diabetes Mellitus, hypertension, dyslipidemia, coronary artery disease, asthma, and cancer. Using backwards selection, a reduced model was developed, identifying age (OR 1.053, 95% CI 0.097–1.07, p < 0.001), hypertension (OR 2.59, 95% CI 1.46–4.59, p = 0.001), and bronchial asthma (OR 3.7176, 95% CI 1.24–11.12, p = 0.018) as significant predictors of long COVID incidence. A significant positive correlation was observed between the symptomatic burden and the number of individual comorbidities.

**Conclusions:**

The significant presence of long COVID at 12 weeks among non-hospitalised patients underscores the importance of post-recovery follow-up to assess for the presence of long COVID. The predictive risk score proposed in this study may help identify individuals at risk of developing long COVID. Further research is needed to understand the impact of long COVID on patients’ quality of life and the potential role of tailored rehabilitation programs in improving patient outcomes.

**Supplementary Information:**

The online version contains supplementary material available at 10.1186/s12879-023-08600-6.

## Background

The vaccination, primary infections and the resultant hybrid immunity in society aided in ending the COVID-19 pandemic. The WHO has rightly urged to shift the focus to the long-term aspects of COVID-19 which includes preventive aspects like vaccination and capacity building and clinical aspects like long term complications associated with COVID-19 including long COVID [1]. Inadequacy in recognising and responding to the long COVID could potentially result in same becoming a significant public health crisis [2]. Long COVID is broadly defined as signs, symptoms, and conditions that continue or develop after initial COVID-19 or Severe Acute Respiratory Syndrome-coronavirus-2 (SARS-CoV-2) infection. The signs, symptoms, and conditions are present four weeks or more after the initial phase of infection; may be multisystemic; and may present with a relapsing– remitting pattern and progression or worsening over time, with the possibility of severe and life-threatening events even months or years after infection [3, 4]. Long COVID encompasses not only the persistence of the symptoms but also a myriad of new onset symptoms, including constitutional, cardiorespiratory, gastrointestinal, musculoskeletal, neurologic, psychosocial and acute thrombotic events, among others, and runs a variable course with varying severity. The post-COVID symptoms are seen even among patients with asymptomatic or mild infections.

The factors attributed to the development of the long COVID include persistent inflammation incited by the viral particles, compounded by the inability of the body to clear the dead virus particles completely. Post viral inflammatory state is described in multiple viral infections, including dengue, influenza and the Epstein-Barr Virus [5]. In comparison, long COVID extends for a substantial duration with the potentially significant impairment of functions leading to a compromised quality of life. Although more than 200 symptoms of varying severity have been linked to the long COVID, common symptoms include fatigue, shortness of breath, and cognitive dysfunction. However, there needs to be more concurrence on the exact symptomatology. Long COVID is not static, but a dynamic entity as the risk of developing the same can be modified by factors such as variants, vaccination and pre-existing comorbidities. The changing nature of the illness warrants continuous observation to recognise the clinical patterns for better characterisation of long COVID.

With the current relaxation in COVID-19 protocols aimed at decreasing viral transmission, the laxity will lead to people opting for conservative measures without testing for aetiology during the symptomatic phase of the viral syndrome [6]. The consequence would be borne by a subset of patients with an increased risk of developing long COVID. Characteristics of the disease which might predict long COVID have not been clearly defined in the Indian setting; attempts to determine the same could represent early milestones for characterising the syndrome and identify the key areas requiring allocation of resources and further research. While it has been proven that vaccination prevents severe COVID-19 [7, 8], individuals may still be infected with the virus, and they may suffer from asymptomatic to mild infections, known as ‘breakthrough infections’. A study from Eastern India found that the proportion of long COVID-19 among patients who had the Omicron variant was considerably lower than those who were infected with the Delta variant [9].

While the existing data on long COVID has been focused on hospitalised individuals, there needs to be more data on real-world lower middle-income nation settings in the post-vaccination era where the majority of the infected individuals undergo treatment in home quarantine [10]. Long-COVID symptoms can be debilitating, significantly affect the quality of life, and presumably can lead to stress on healthcare systems in future years [11]. The resulting chronic syndromes of pain, fatigue and respiratory inflammation may also incite mental health issues in patients. It thus becomes vital to recognise the clinical profile of vaccinated patients at risk for long-COVID to find ways to mitigate these risk factors in the future.

We aim to describe the incidence of long COVID among post-ChAdOx1 n-Cov-2 vaccine breakthrough infections in a South Indian cohort who tested positive for COVID-19 during the period from October 2021 to December 2021 and also to characterise the clinical features along with risk profiling of the patients with long COVID.

## Methods

### Study design and setting

This was a single-centre hospital-based prospective observational study conducted at a 1350 bedded academic tertiary care referral centre in South India from October 2021 to December 2021, after the second wave of the pandemic that began in April 2021, in the country. The COVID-19 variant predominant in circulation in India during our study period was delta as per the data from INSACOG (https://dbtindia.gov.in/insacog ) [12]. The hospital was a major regional medical hub, catering for mild to severe COVID-19 cases with a dedicated isolation facility that comprised intensive care units and non-intensive care locations for inpatients. The hospital also had an exclusive COVID-19 clinic to formulate the plan of management for cases that required only isolation measures at their residence.

The study was approved by the Institutional Ethics Committee and informed signed consent was obtained from all subjects before enrolling.

### Study Population

The study population comprised adult patients (> 18 years) who had a confirmed COVID-19 diagnosis through positive Reverse Transcriptase-Polymerase Chain Reaction (RT-PCR) tests and were either admitted to the inpatient COVID-19 ward and subsequently got discharged from the hospital or underwent isolation at their respective homes. These subjects were screened for the inclusion criteria that specified the administration of at least a single dose of vaccination to identify breakthrough COVID-19 infections. Patients who were younger than 18 years old, pre/post-surgical patients, patients who were transferred to a non-COVID-19 inpatient location for continuation of care for indications other than COVID-19, and end-of-life care were excluded from the study.

### Data collection

A dedicated healthcare worker was trained to inquire about the symptoms post breakthrough infections using a specially tailored questionnaire (Additional file 1) on week 2, week 6 and week 12 after the patient was confirmed to have a negative COVID-19 test. The prospective survey recorded data on the demographics and pre-existing comorbidities of the patient, neuropsychological manifestations of long COVID (fatigue, anxiety, depression, dyspnoea), other somatic manifestations (fever, headache, cough, myalgia, arthralgia, chest pain etc.), presence of any superseding infections during the period of COVID-19 positivity which may have had a compounding effect in the manifestation of long COVID, oxygen requirement during the period of hospitalisation, work and functional status after testing negative for COVID-19.

The functional status was assessed longitudinally at 2, 6 and 12 weeks using the Modified Oswestry Scale [13–16]. Based on the modified Oswestry Disability Index (ODI) score, patients were categorized from A (minimal disability) to E (bed bound or exaggerating symptoms) with scoring intervals as follows: A (Minimal disability: 0 to 20), B (Moderate disability: 21 to 40), C (Severe disability: 41 to 60), D (Cripple, pain impinging on all aspects of life: 61 to 80) and E (Bed-bound or exaggerating symptoms: 81 to 100).

The vaccination status of the individual was considered to be complete if the subject had received both of the two scheduled COVID-19 vaccine doses as mandated by the Ministry of Health and Family Welfare (MoHFW), Government of India, while partial vaccination status indicated the receipt of a single first vaccine dose. Booster doses were not available to the general public, at the time of the study, and were hence not considered in determining the immunisation status of the subject.

The data was collected over a telephonic conversation for consenting patients, using the questionnaire as a template, at the aforementioned time intervals. This was then tabulated into a database, after which the clinical details of each patient were cross-verified from the electronic medical records, present in the hospital information systems. Once verified, the data was submitted for statistical analysis.

### Outcomes

The primary outcome was to estimate the prevalence of long COVID in our study cohort of breakthrough infections. In our study, long COVID was defined by the presence of new or persistent symptoms at or beyond 6 weeks’ after the initial SARS-CoV-2 infection [17, 18]. As per the NICE guidelines, the term ‘Long COVID’ encompasses both the symptomatic (4–12 weeks) and post COVID syndrome (> 12 weeks) [19].

### Statistical analysis

The baseline characteristics of the study cohort were summarized using descriptive statistics. The key indicators were expressed in mean and standard deviation for continuous variables and in terms of frequency and percentages for categorical variables. Differences in categorical baseline characteristics between the patients who developed post COVID symptoms and those who did were tested by Chi square test for independence. Multiple logistic regression was used to estimate the influence of the patient characteristics on the chance of developing long COVID. A propensity score for predicting long COVID was developed by backward step-down variable deletion based on AIC values [20]. The score is represented by a nomogram [21]. To study the process of the variable process further, variance influence factors are calculated for the possible predictors.

The Average AUC (Area Under the ROC Curve) values were reported from 1000 replications of the 10-fold cross-validation procedure. The final propensity score was obtained by refitting the model with the most frequently selected variables using the full dataset. The overall model performance was assessed by Nagelkerke’s R2 and the Brier score, the discriminative ability by the C concordance statistic and by Somers Dxy rank correlation, and model calibration by Hosmer-Lemeshow test [22]. Significances of differences in individual long COVID symptoms depending on the propensity score were tested with 2 sample-independent t-tests. The dependency of the symptom burden based on the propensity score was estimated using linear regression models with natural cubic splines; similarly, the probability for a decrease in the functional score from 2 to 12 weeks was estimated using logistic regression with natural splines. Regression lines with 95% confidence bands were plotted and the significance of these relationships was tested using F-tests. All statistical analysis for the study was performed in R version 4.3.1, R Foundation for Statistical Computing, Vienna, Austria [23]. P < 0.05 was considered to be statistically significant.

## Results

### Baseline demographics

Among the 414 patients followed up in the study, 51.2% (n = 212) were found to be females and the majority of the patients belonged to 18–39 years of age (45.2%, 187) (Table 1). Diabetes mellitus (23.18%) and hypertension (26.08%) among were found to be the predominant co-morbidities in the study cohort while 49% (n = 204) patients did not report any co-morbidity. Hospitalization during COVID-19 was reported among 5.3% (n = 22) patients. The proportion of patients who completed the full vaccination schedule of the first and second doses was found to be 96.5% (n = 394).

**Table 1:** Demographic data and COVID-19 characteristics of patients. Age is summarized by mean and SD. The remaining categorical variables are summarized by sample sizes and percentages in each level

Characteristics	Overall (N = 414) (%)
Age, years (mean ± SD)	44.41 ± 17.7
18–39 years	187 (45.2%)
40–59 years	127 (30.7%)
Above 60 years	100 (24.2%)
Gender	
Male	202 (48.8%)
Female	212 (51.2%)
Co-morbidities	
Type II Diabetes Mellitus	96 (23.18%)
Hypertension	108 (26.08%)
Dyslipidemia	40 (9.66%)
Hypothyroidism	33 (7.97%)
Chronic Liver Disease	34 (8.21%)
Chronic Kidney Disease	13 (3.14%)
Bronchial Asthma	20 (4.83%)
Chronic Obstructive Pulmonary Disease	16 (3.86%)
Coronary Artery Disease	10 (2.41%)
Cancers	16 (3.86%)
Number of co-morbidities	
No co-morbidities	204 (49.2%)
Single co-morbidity	106 (25.6)
Two co-morbidities	54 (13%)
3 co-morbidities and above	50 (12%)
COVID-19 events	
Hospitalization during COVID-19	22 (5.3%)
Hospitalization post COVID	3 (0.72%)
Oxygen Requirement post COVID	1 (0.24%)
Vaccination status	
Fully vaccinated	395 (96.5%)
Partially vaccinated	19 (4.5%)

### Symptom profile

The presence of long COVID symptoms reported among the study cohort beyond 6 weeks post-COVID infection was observed among 164 patients (39.6%, 95% CI 34.9% − 44.3%). Figure 1 A illustrates the post-COVID symptomatic distribution among the study cohort at 2, 6 and 12 weeks. The proportion of patients with long COVID symptoms at the end of 6 and 12 weeks was found to be 38.9% (n = 161) and 10.1% (n = 42) respectively. 39 patients (9.4%) had persistent symptoms from 6 to 12 weeks post-COVID, while only 3 patients recorded a new onset of long COVID symptoms at the end of 12 weeks. 29.4% (n = 122) of patients had symptoms at 6 weeks without symptoms persisting up to 12 weeks of testing negative for COVID-19. The percentage reduction in symptoms in 12 weeks with respect to 2 weeks was minimal for dyspepsia (81%), followed by fatigue (86.5%). Symptoms such as sleep abnormalities, diarrhoea, weight loss, constipation and fever disappeared completely by 12 weeks.

**Fig. 1A:**
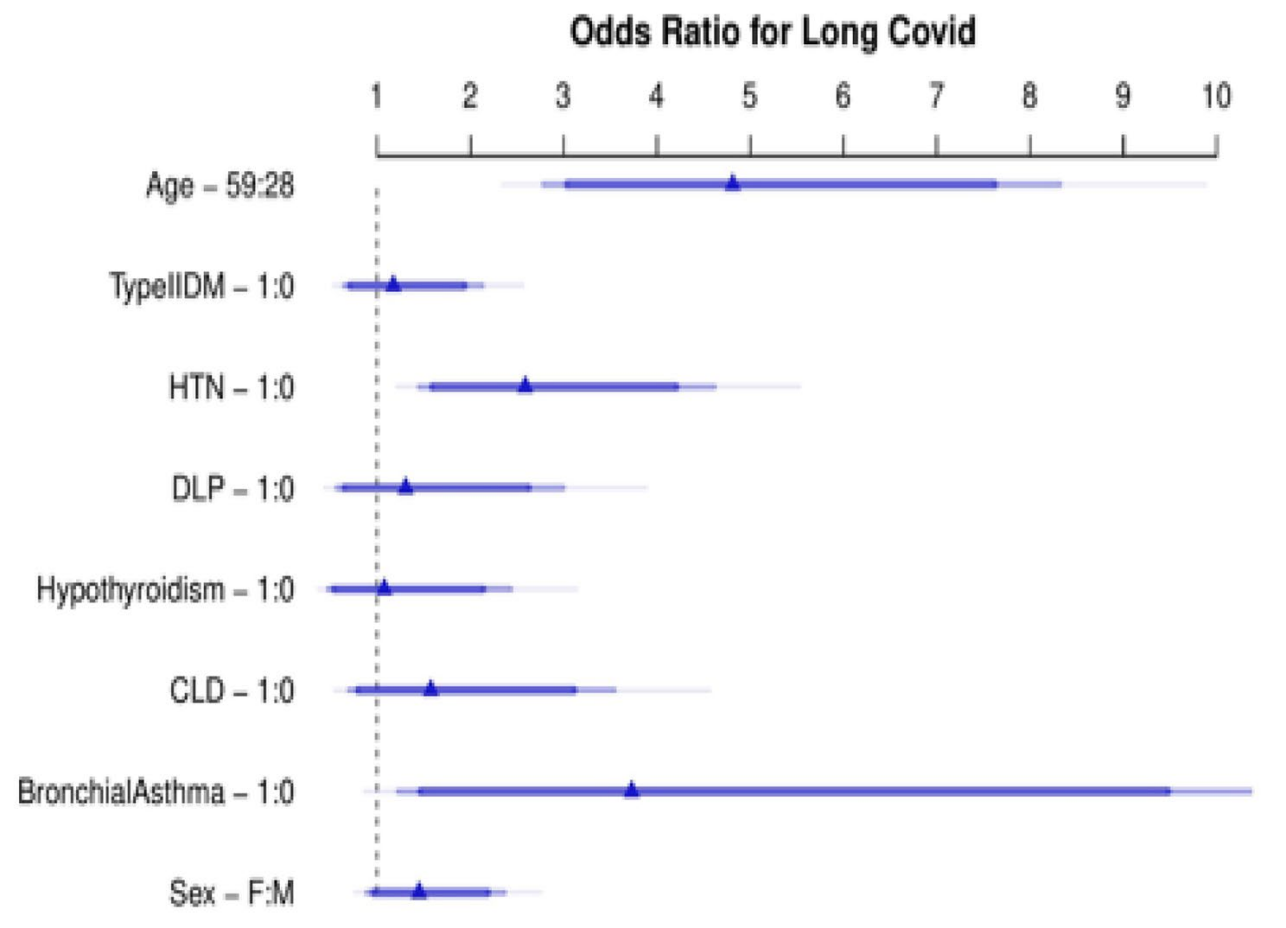
Long COVID symptomatic profile at weeks 2, 6 and 12 weeks

**Fig. 1B:**
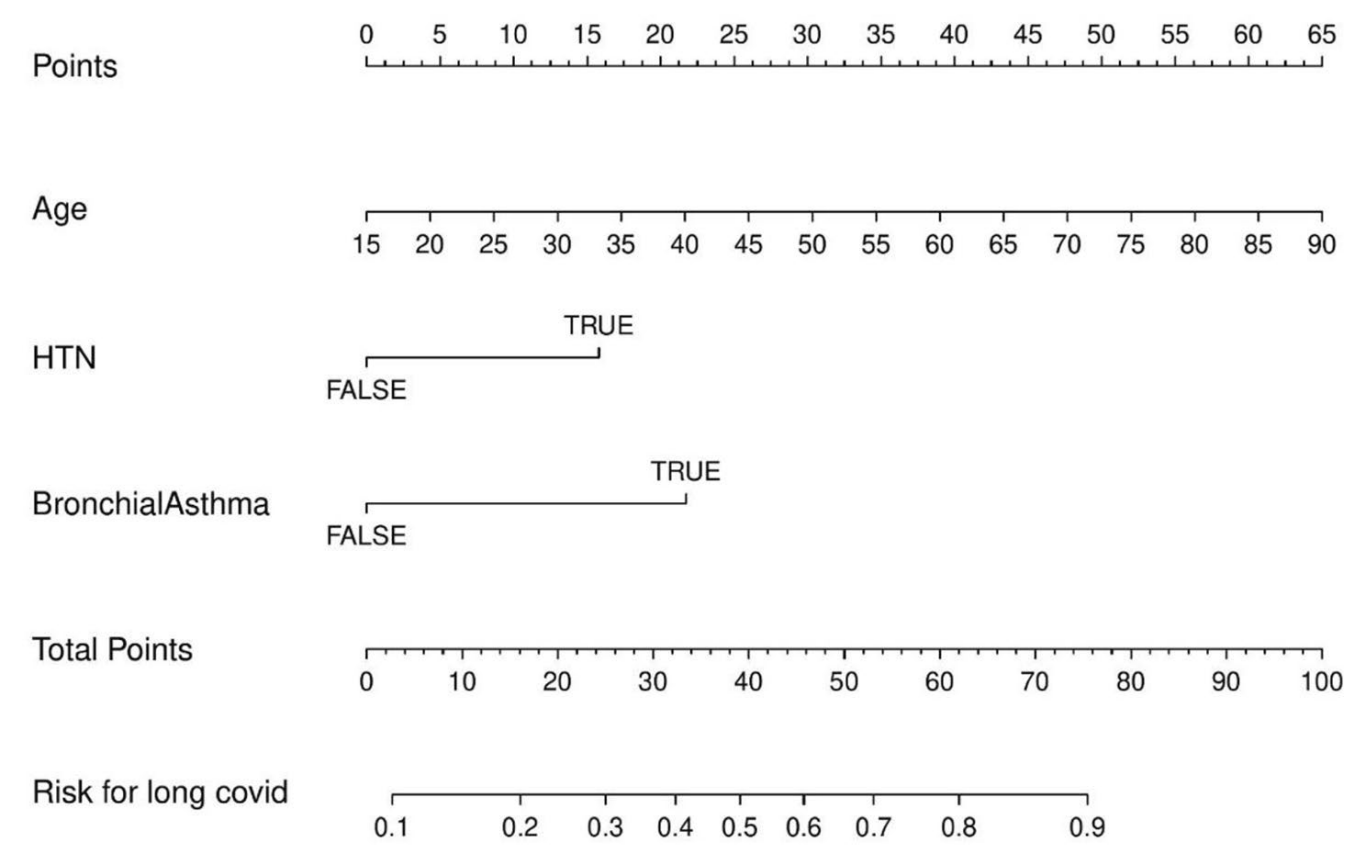
Organ system wise distribution of long COVID symptoms at 2, 6 and 12 weeks

Organ-wise symptomatic profile revealed that 79 patients (19.1%) were found to have constitutional symptoms (fatigue, fever, weight loss), 81 patients (19.5%) had respiratory symptoms (cough, dyspnoea, chest pain), 47 patients (11.3%) reported gastrointestinal symptoms (dyspepsia, bowel movement disturbances), 40 patients (9.6%) were found to have musculoskeletal symptoms (myalgia/arthralgia) and 72 patients (17.3%) were found to have neuropsychiatric symptoms (headache, paraesthesiae, mood disturbances and sleep abnormalities). Figure 1B describes the organ-wise symptomatic profiles at weeks 2, 6 and 12. While gastrointestinal and constitutional symptoms recorded the lowest percentage reduction (85.9% and 87.1%, respectively) within 12 weeks post-COVID infection, musculoskeletal and neuropsychiatric symptoms registered the highest percentage reduction at 97% each, in the same period.

The post-COVID functional status, which has been depicted in Table 2, was assessed by the Modified Oswestry Scale, first described in 2001 in *Physical Therapy* and is a modification of the original scale developed by Fairbank et al. in 1981^[12–15]^. At 12 weeks, the moderate disability (B) was observed to be 4% in the whole cohort. Functional status changes from E to D was 0% and 0% from weeks 2–6 and weeks 6–12, respectively; from D to C was 1.21 and 0% from weeks 2–6 and weeks 6–12, respectively; from C to B was 4.35% and 0.97% from weeks 2–6 and weeks 6–12, respectively and 15.22–4.11% from weeks 2–6 and weeks 6–12, respectively.

**Table 2:** Functional status assessed by Modified Oswestry Scale

Functional Status	2 Weeks	6 Weeks	12 Weeks
A	76.57%	91.79%	95.89%
B	18.12%	7.25%	4.11%
C	4.11%	0.97%	0.00%
D	1.21%	0.00%	0.00%
E	0.00%	0.00%	0.00%

### Risk factors for the presence of long COVID

In a univariable analysis, we compared factors that were associated with long COVID incidence in the study cohort. Presence of long COVID was found to be significantly higher among patients above 65 years of age at 74% in comparison to the long COVID presence at 32% among those below age 65 (p < 0.001). 64% of patients with Type II Diabetes Mellitus (DM) who reported long COVID symptoms relative to 36% without diabetes (p < 0.001). Hypertension (HTN) was significantly associated with the presence of long COVID with 72% of hypertensive patients reporting long COVID symptoms in comparison to 28% without hypertension (p < 0.001). 70% of patients with dyslipidemia reported significantly high long COVID incidence compared to 30% without dyslipidemia (p < 0.001). Presence of long COVID was also significantly high among patients with coronary artery disease (CAD) with 80% of patients with the condition reporting long COVID (p = 0.017). 70% of patients with asthma reported long COVID (p = 0.008). Long COVID incidence was significantly high among cancer patients with 70% of the patients reported long COVID (p = 0.04). Other comorbidities including sex, hypothyroidism, chronic liver disease (CLD), chronic kidney disease (CKD), and chronic obstructive pulmonary disorder (COPD) were not found to be significantly associated with long COVID (Table 3).

**Table 3:** Association of baseline characteristics and COVID-19 treatment with presence of long COVID symptoms. Variables are summarized by sample sizes and percentages, stratified by the presence and absence of COVID. Chi square test for independence were used to calculate the p-values for each of the variables

	Fully Vaccinated cohort				
Variables	Presence of long COVID (n = 164)	Absence of long COVID (n = 250)	OR (95%CI)	p value	
Age > 65	57 (74%)	20 (26%)	6.13(3.5-10.71)	< 0.001*	
Age < 65	107 (32%)	230 (68%)			
Male	84 (42%)	118 (58%)	1.17 (0.79–1.74)	0.242	
Female	80 (37%)	132 (63%)			
Type II DM	59 (64%)	33 (36%)	3.69 (2.27-6)	< 0.001*	
Hypertension	78 (72%)	30 (28%)	6.65 (4.08–10.85)	< 0.001*	
DLP	28 (70%)	12 (30%)	4.08(2.01–8.29)	< 0.001*	
Hypothyroidism	17 (52%)	16 (48%)	1.69 (0.83–3.45)	0.193	
CLD	21 (62%)	13 (38%)	2.68 (1.3–5.51)	0.01*	
CKD	8 (40%)	12 (60%)	1.02 (0.41–2.54)	0.613	
Asthma	14 (70%)	6 (30%)	3.8 (1.43–10.09)	0.008*	
COPD	7 (44%)	9 (56%)	1.19 (0.44–3.27)	0.797	
CAD	8 (80%)	2 (20%)	6.36 (1.33–30.33)	0.017*	
Cancer	7 (70%)	3 (30%)	3.72 (095-14.58)	0.04*	

*Signifies statistically significant numbers

**DLP = Dyslipidemia CLD = Chronic Liver Disease CKD = Chronic Kidney Disease COPD = Chronic Obstructive Pulmonary Disorder CAD = Coronary Artery Disease**

**Fig. 2:**
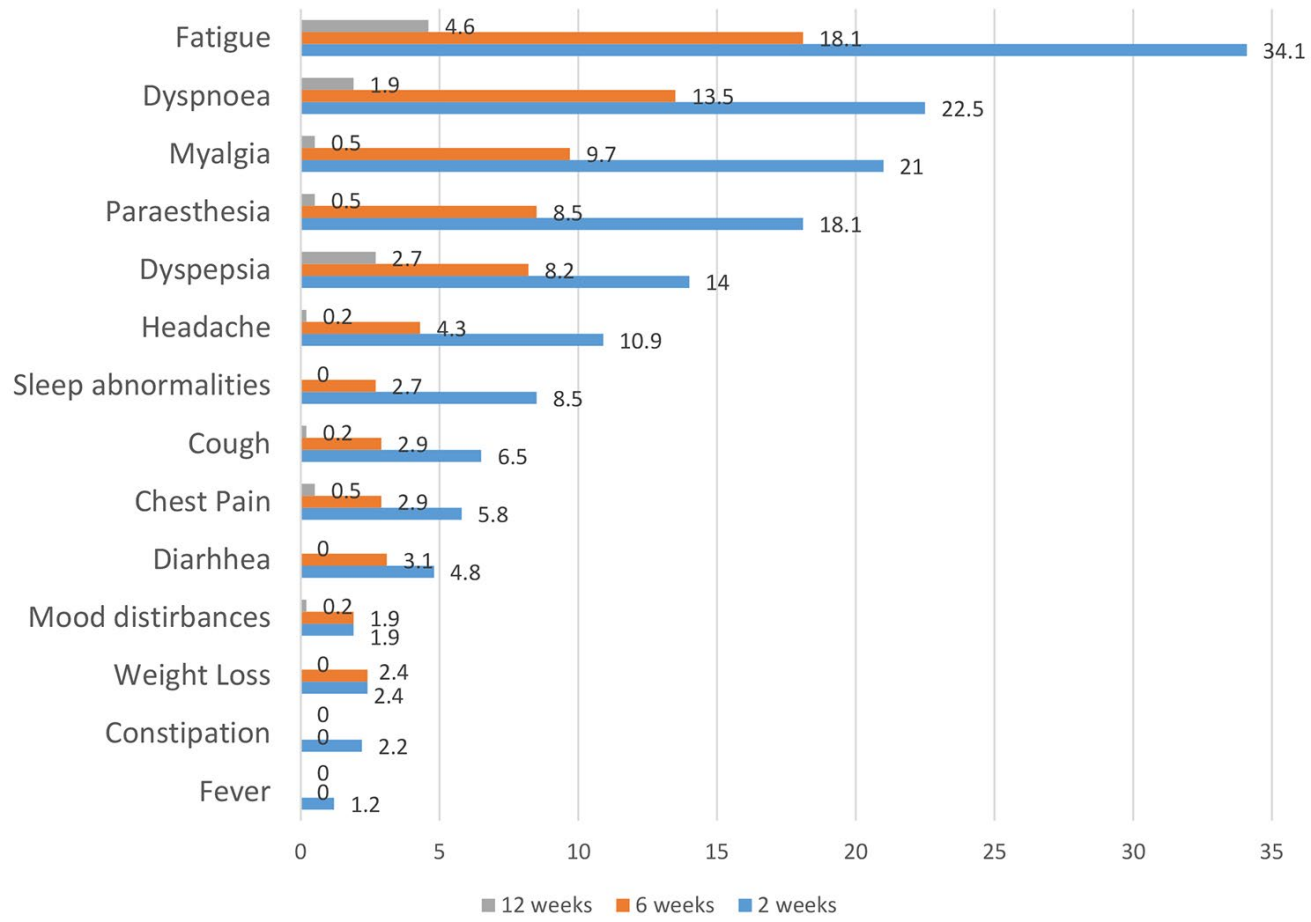
Distribution of number of long COVID symptoms among the number of comorbidities

The symptomatic burden defined as the number of symptoms per individual was observed to have a significant positive correlation with the number of comorbidities for an individual (r = 0.404, p < 0.001) (Fig. 2).

### Development of propensity score for the risk of long COVID incidence

The logistic regression model for developing long COVID depending on patient characteristics has been depicted in Fig. 3A (C statistics – 80.9%). Using backwards selection, non-significant terms were removed from the model leading to the reduced model comprising of age (OR 1.053, 95% CI 0.097–1.07), p < 0.001), hypertension (OR 2.59, 95% CI 1.46–4.59, p = 0.001)) and bronchial asthma (OR 3.7176, 95% CI 1.24–11.12, p = 0.018) to be significant predictors of long COVID incidence were included in development of of propensity score. The model performance was measured as Nagelkerke’s R2 0.339, Brier score 0.176, C statistic 0.809, Somers Dxy 0.610, and Hosmer-Lemeshow p-value of 0.05 (Supplementary table S1). The cross-validation value of the AUC was 80.3%. The nomogram for long COVID from the variables in the reduced model is shown in Fig. 3B. The propensity score and probabilities from this nomogram are shown in Table 4.

**Table 4:** Propensity scoring calculation and probabilities in percentages.

SCORE	PROBABILITY FOR LONG COVID (%)
3	10%
16	20%
25	30%
32	40%
39	50%
46	60%
53	70%
62	80%
75	90%

Propensity score = 0.867 * (Age − 15) + Hypertension + Bronchial Asthma#.

^#^Age in years to be given in the equation.

^#^If hypertension is present, the scoring value is 16.

^#^Similarly, the scoring value for Bronchial Asthma is 22.

**Fig. 3A:**
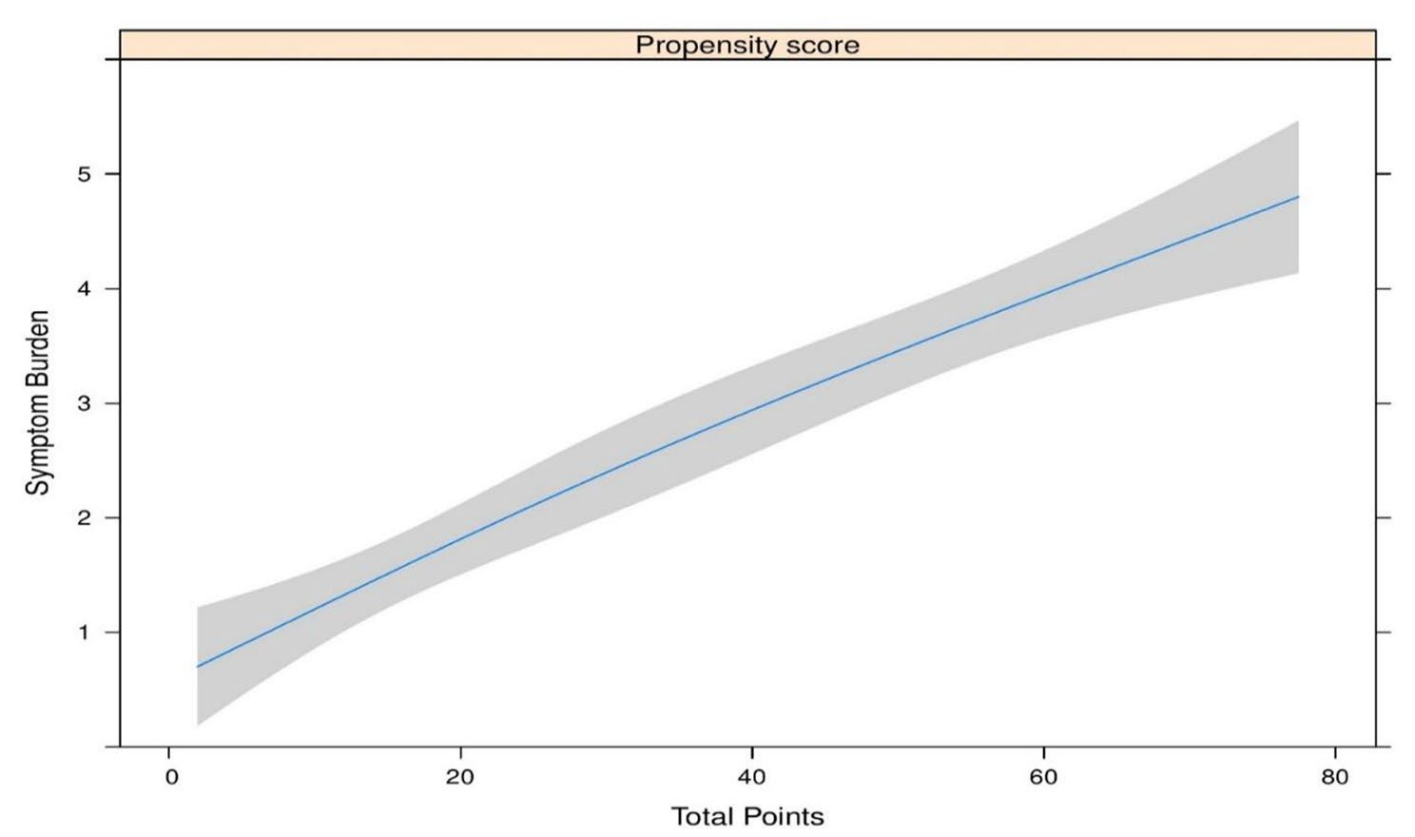
Logistic regression model for predicting long COVID incidence

**Fig. 3B:**
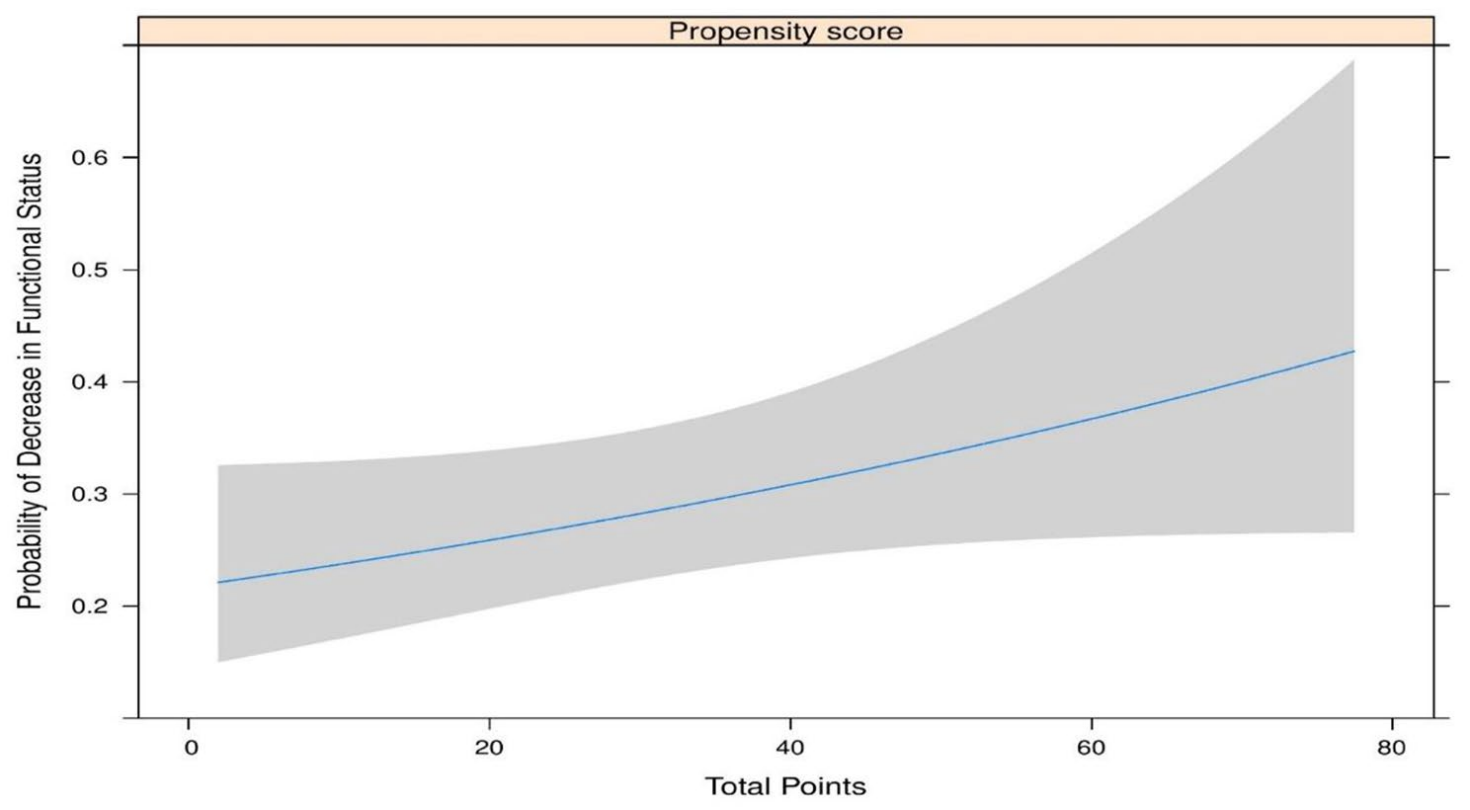
Nomogram representation of the prediction model for long covid incidence. The prediction is based on the values of variables age, hypertension and bronchial asthma. Each variable is represented by a line in the nomogram. To make a prediction for a new patient, first determine the known values of the variables on the nomogram. Then follow the lines for each variable and note the corresponding point on the first line in the nomogram. Add these points for all three variables. Finally, to make the prediction, add these points and then compare the position of the resulting total points on the next to last line, with the predicted probability on the last line

Table 4 details the differences in propensity scores for individual long COVID symptoms, indicating significant difference in the mean propensity risk scores between the presence and absence of majority of long COVID symptoms. The propensity score is significantly associated with the reduction of functional status from 2 to 12 weeks as in Fig. 4A (p < 0.05). The relationship between propensity scores and symptom burden is shown in Fig. 4B (p < 0.01).

**Fig. 4A:**
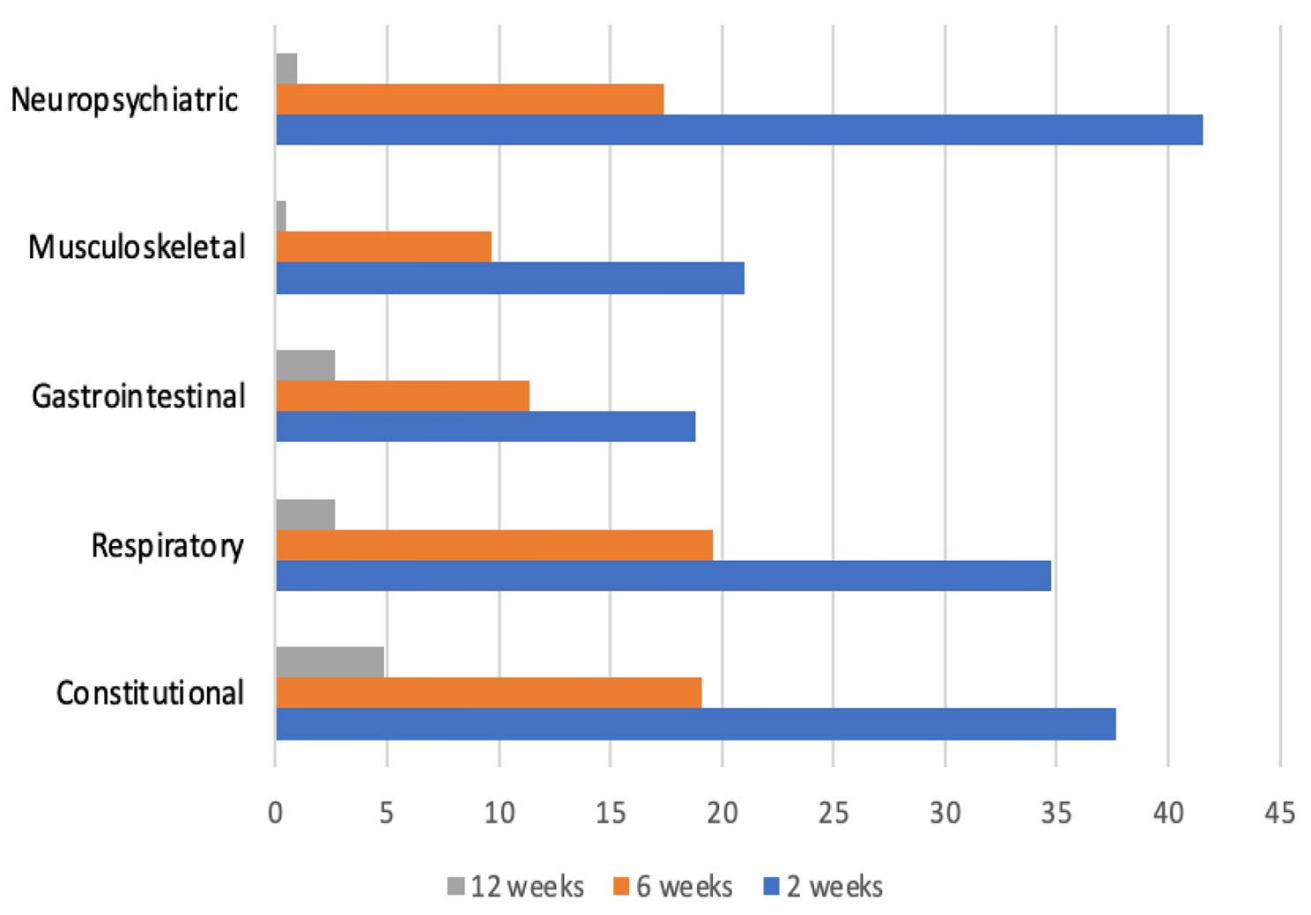
Association of propensity risk score for Long COVID incidence with symptom burden

**Fig. 4B:**
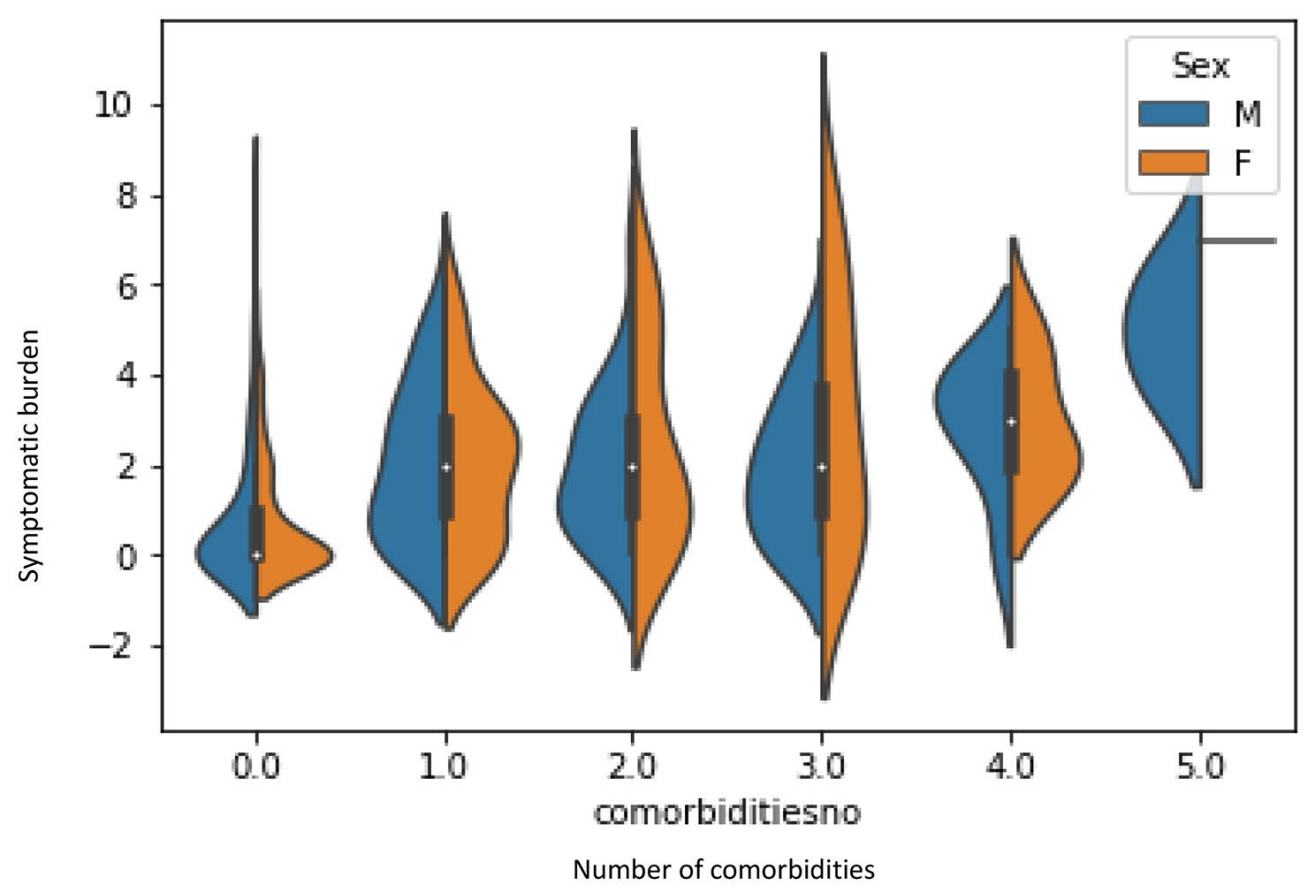
Association of propensity risk score for Long COVID incidence with functional status

## Discussion

Our prospective observational study among post recombinant ChAdOx1-S/nCoV-19 vaccine breakthrough COVID-19 infections in a South Indian cohort revealed the incidence of long COVID to be clinically important at 38.9% (161) and 10.1% (42) at 6 and 12 weeks respectively. Regression analysis in our study identified age, bronchial asthma and hypertension to be significant risk factors predisposing to the development of long COVID. The identified risk factors were used to develop a predictive scoring system for the incidence of long COVID based on propensity-weighted risk scoring which demonstrated a significant linear correlation with symptom burden and functional score.

Our study represents an effort to predict the incidence of long COVID among predominantly non-hospitalized patients from an Indian setting where long COVID is relatively unexplored with scarce data. The major long COVID symptoms reported at the end of 6 weeks were fatigue (18.5%), followed by dyspnoea (13.1%) and myalgia (9.7%). At 12 weeks, fatigue remained to be the pre-dominant long COVID symptom at 4.6%, followed by dyspepsia (2.7%) and dyspnoea (1.9%). Global estimates reportedly indicated a pooled prevalence of 0.43 or 43% (95% CI 0.39–0.46) with regional prevalence estimates observed to be higher among Asians (0.51), in comparison to Europeans (0.44) and United States (0.31) [24]. Long COVID incidence at 12 weeks in our study is observed to be relatively similar to the earlier data in November 2022 from Office for National Statistics (ONS), UK reporting an estimate of 12% of patients self-reporting the symptoms, where fatigue was the major symptom at 8.4%, followed by difficulty in maintaining concentration (5.4%) and shortness of breath (5.04%) [25]. In a prior study from an Indian setting, the most common symptoms reported were fatigue (22.60%), cough (9.60%) and myalgia (7.54%) [26]. However, this study portrays the result from a hospitalized cohort with the findings of the study constrained by a short follow-up period of 30 days and reports of vaccination status being unavailable.

Our study cohort was a predominantly non-hospitalized cohort with only 5% requiring hospital stay during the period of COVID-19 infection. The risk of developing long COVID was observed to be higher in hospitalized COVID-19 patients in comparison to non-hospitalized patients. The estimated pooled prevalence for long COVID was found to be higher among hospitalized cohort (0.54) relative to the non-hospitalised cohort (0.34), potentially suggesting the higher probability of long COVID incidence proportional to the severity of COVID-19 infections as indicated by hospitalization. Our previous study among unvaccinated hospitalized COVID-19 patients during early 2021 demonstrated the presence of at least one persistent long COVID symptom at the sixth week of discharge in 60.8% of the study cohort. The currently observed reduction in the incidence of Long COVID at 38.9% in our study probably indicates the impact of hospitalization and the potential protective effects of vaccination on the development of long COVID. The association of the vaccine with reduced risk or odds of long COVID has been evidenced by a meta-analysis that went on to suggest that two doses could be more effective than one dose [24, 27]. However, the decreasing rates of long COVID incidence during different pandemic wave periods have been observed in an Italian study among a non-hospitalized cohort of healthcare workers indicating the influence of prevalent variants during the waves on the development of long COVID [28]. Similarly, there could also be a possible impact of the variants during each wave for the reduction in the long COVID incidence observed.

Several predisposing risk factors including pre-existing co-morbidities, the severity of COVID-19 and demographic characteristics such as age and gender have been identified as predictors of long COVID in various studies [27, 29, 30]. The Italian study has identified the elderly age group, obesity and airway disease to be significantly associated with long COVID risk. Similarly, our study revealed age, hypertension and bronchial asthma to be significant predictors of long COVID in multivariate regression analysis. Long COVID is not considered a single dimensional disease with a prolonged course but rather as an episodic disease with fluctuations and varying severity [31]. Hence, a predictive risk score of long COVID should be able to identify not only the presence and absence of long COVID but also indicate the severity of long COVID.

The predictive risk scoring developed based on the significant risk factors identified in our study demonstrated a positive correlation between symptom burden and functional disability of the patient.

Recognition of long COVID and its severity has important public health consequences as the resultant functional disability can impact the ability to perform daily living activities and employment-related duties of an individual resulting in socioeconomic consequences. Such individuals could benefit from a long term follow up approach resulting in the early pick up of the debilitation and focused rehabilitation. The societal impact of long COVID can be reduced by preventive measures such as mass vaccination in community settings [32–34]. This needs to be re-iterated in the context of increasing vaccine hesitancy.

### Limitations

The recruitment of COVID-19 patients into the study was limited to the period of a single pandemic wave, limiting the generalizability of the findings to other variants. However, the risk factors and their predictors would remain largely applicable as extrapolated from other studies. Considering the two major vaccines were ChAdOx1 and Covovax in the Indian context, our study observations were limited to only breakthrough infections post-ChAdOx1 vaccination. During the period, the vaccination rates in the community were high with 96.5% of our cohort being recipients of 2 doses of vaccination. The lack of an unvaccinated arm in our study limits our interpretation owing to the lack of a head-to-head comparison regarding the impact of vaccination on long COVID. Our study cohort was pre-dominantly non-hospitalized patients and hence it may not apply to hospitalized patient cohorts. Another limitation is lack of in-person interview and examination during follow-ups.

A longitudinal follow-up of our cohort to assess the persistence or recurrence of the long COVID symptoms would be beneficial as it is a currently evolving post-infectious syndrome that is not completely characterized. We believe a prospective validation of the score for different variants at different periods would add to the generalizability of the predictive scoring system.

## Conclusions

The prospective observational study among predominantly non-hospitalized patients with breakthrough COVID-19 infections revealed a significant presence of long COVID at 12 weeks warranting a continuous follow-up of the COVID-19 recovered patients for early identification and appropriate management. The predictive risk scoring based on significant risk factors could identify the patients at risk for developing long COVID to provide focused attention and continual care.

### Electronic supplementary material

Below is the link to the electronic supplementary material.

Supplementary Material 1. Additional file 1: Pdf. A follow-up questionnaire to assess the post-covid-19 manifestations in a cohort of patients with breakthrough infections who have recovered from covid-19 at a tertiary care hospital

## Data Availability

The datasets used and/or analyzed during the current study are available from the corresponding author on reasonable request.

## List of abbreviations

SARS-CoV-2: Severe Acute Respiratory Syndrome-coronavirus-2
RT-PCR: Reverse Transcriptase-Polymerase Chain Reaction
OSI: Oswestry Disability Index
MoHFW: Ministry of Health and Family Welfare
WHO: World Health Organization
AUM: Area Under the Curve
Type-2 DM: (Type-2 Diabetes Mellitus)
HTN: Hypertension
CAD: Coronary Artery Disease
CLD: Chronic Liver Disease
CKD: Chronic Kidney Disease
COPD: Chronic Obstructive Pulmonary Disease
ONS: Office for National Statistics
