# Supplementary material for: Characterization and predictive risk scoring of long COVID in a south indian cohort after breakthrough COVID infection; a prospective single centre study
Source: BMC Infect Dis. 2023 Oct 9;23:670. doi: 10.1186/s12879-023-08600-6 (PMC10563355; doi:10.1186/s12879-023-08600-6)
Supplement: Supplementary file 1 — Supplementary Material 1. Additional file 1: Pdf. A follow-up questionnaire to assess the post-covid-19 manifestations in a cohort of patients with breakthrough infections who have recovered from covid-19 at a tertiary care hospital [file 12879_2023_8600_MOESM1_ESM.pdf]

Supplementary table S1: Variance inflation factors (VIF) of the possible predictors for the creation of the prediction model.

Predictor	VIF
Age	1.498
Hypertension	1.212
Type-2 Diabetes Mellitus	1.201
Chronic Obstructive Pulmonary Disorder	1.118
Female	1.108
Dyslipidemia	1.076
Cancer	1.067
Coronary Artery Disease	1.039
Chronic Liver Disease	1.038
Chronic Kidney Disease	1.032
Hypothyroidism	1.028
Bronchial Asthma	1.013
